# Factors associated with tuberculosis infection, and with anti-mycobacterial immune responses, among five year olds BCG-immunised at birth in Entebbe, Uganda

**DOI:** 10.1016/j.vaccine.2014.12.015

**Published:** 2015-02-04

**Authors:** Swaib Abubaker Lule, Patrice A. Mawa, Gyaviira Nkurunungi, Margaret Nampijja, Dennison Kizito, Florence Akello, Lawrence Muhangi, Alison M. Elliott, Emily L. Webb

**Affiliations:** a MRC/UVRI Uganda Research Unit on AIDS, PO Box 49, Entebbe, Uganda; b Entebbe Hospital, PO Box 29, Entebbe, Uganda; c London School of Hygiene & Tropical Medicine, Keppel Street, London WC1E 7HT, UK

**Keywords:** Tuberculosis, HIV, Helminth, Pregnancy, Bacille Calmette–Guerin, Crude culture filtrate protein

## Abstract

**Background:**

BCG is used widely as the sole licensed vaccine against tuberculosis, but it has variable efficacy and the reasons for this are still unclear. No reliable biomarkers to predict future protection against, or acquisition of, TB infection following immunisation have been identified. Lessons from BCG could be valuable in the development of effective tuberculosis vaccines.

**Objectives:**

Within the Entebbe Mother and Baby Study birth cohort in Uganda, infants received BCG at birth. We investigated factors associated with latent tuberculosis infection (LTBI) and with cytokine response to mycobacterial antigen at age five years. We also investigated whether cytokine responses at one year were associated with LTBI at five years of age.

**Methods:**

Blood samples from age one and five years were stimulated using crude culture filtrates of *Mycobacterium tuberculosis* in a six-day whole blood assay. IFN-γ, IL-5, IL-13 and IL-10 production was measured. LTBI at five years was determined using T-SPOT.TB^®^ assay. Associations with LTBI at five years were assessed using multivariable logistic regression. Multiple linear regression with bootstrapping was used to determine factors associated with cytokine responses at age five years.

**Results:**

LTBI prevalence was 9% at age five years. Only urban residence and history of TB contact/disease were positively associated with LTBI. BCG vaccine strain, LTBI, HIV infection, asymptomatic malaria, growth z-scores, childhood anthelminthic treatment and maternal BCG scar were associated with cytokine responses at age five. Cytokine responses at one year were not associated with acquisition of LTBI by five years of age.

**Conclusion:**

Although multiple factors influenced anti-myocbacterial immune responses at age five, factors likely to be associated with exposure to infectious cases (history of household contact, and urban residence) dominated the risk of LTBI.

## Highlights

• Urban residence and history of TB contact/disease were associated with increased risk of latent TB infection at age five years.
• BCG vaccine strain, LTBI, HIV and malaria infections, and anthropometry predict anti-mycobacterial immune responses.
• Helminth infections do not influence response to BCG vaccination.
• Cytokine responses at one year were not associated with LTBI at age five years.

## 1 Introduction

Latent tuberculosis infection (LTBI) is an important reservoir from which new tuberculosis (TB) cases arise. In children, active TB disease results either from reactivation of LTBI or from primary infection acquired from contact with an infectious individual [1]. Lifetime risk of LTBI reactivation is approximately 10% [2]. In endemic settings, Bacille Calmette–Guerin (BCG) is routinely offered to protect infants from TB [3]. Recently, evidence has accrued that BCG may protect against infection with *Mycobacterium tuberculosis*, as well as against TB disease [4,5]. However, the protective efficacy of BCG varies widely [6–10]. Despite advances in immunology, there are no sufficiently validated immune correlates of BCG-induced protection [11] and mechanisms of protection are poorly understood. Accurate information is lacking on risk factors and immunological markers associated with subsequent TB infection or disease among children from developing countries.

Proposed explanations for variation in BCG efficacy include population genetics [10,12], BCG vaccine strains [13–15], environmental mycobacteria exposure [8,14,16], strains of *Mycobacterium tuberculosis* (M.tb) [17], exposure to chronic helminth infections [9,12,18] and nutritional status [10,12].

BCG induces strong interferon gamma (IFN-γ) production, a type 1 immune response essential for protection against M.tb [19,20]. IFN-γ alone may not be sufficient for disease prevention [20], but excessive production of type 2 cytokines may be detrimental [21]. Interleukin-10 (IL-10) is associated with suppression of protective responses [22]. Although BCG induces only minor type 2 responses, these may be sufficient in some individuals to undermine the efficacy of type 1-mediated immunity and cause immune-pathology [23]. Differences predetermined *in utero*, or during the first months of life, may explain the variable protection induced by BCG [8].

In this cohort of children who received BCG at birth, we investigated factors associated with LTBI at age five, factors associated with cytokine responses to BCG at age five and whether cytokine responses at one year were associated with subsequent LTBI. Understanding these relationships may aid the development of new vaccines against tuberculosis, several of which are either recombinant forms of BCG or designed to boost responses primed by BCG [24].

## 2 Methods

### 2.1 Study design and setting

We analysed data from the Entebbe Mother and Baby Study (EMaBS), a randomised double-blinded placebo-controlled trial of anthelminthic treatment in pregnancy and early childhood, conducted in a peri-urban and rural setting by Lake Victoria, Uganda (ISRCTN32849447). EMaBS was established to investigate effects of helminths and their treatment on immune responses to vaccines and on susceptibility to infectious and allergy-related diseases. The design and results of the trial have been reported [25–27]. Briefly, women attending antenatal care at Entebbe hospital were enrolled between April 2003 and November 2005, and randomised to receive single dose albendazole (400 mg) or placebo and praziquantel (40 mg/kg) or placebo in a 2 × 2 factorial design. At age fifteen months, their children were randomised to receive albendazole or placebo quarterly until age five years. In 2008, additional funding was awarded which allowed us to assess children for LTBI at age five.

### 2.2 Study objectives

For this analysis, our primary objectives were to investigate (1) factors associated with LTBI at age five years, (2) factors associated with cytokine response to BCG at age five years, and (3) whether cytokine responses at one year of age were associated with acquisition of LTBI by age five years, among children with documented BCG immunisation in infancy.

### 2.3 Study procedures

Socio-demographic data, blood and stool samples were obtained at enrolment during pregnancy. Children received routine BCG immunisation (with polio immunisation) at birth. The three BCG vaccine strains used were provided by the National Medical Stores according to availability: BCG-Russia (BCG-I Moscow strain, Serum Institute of India, India); BCG-Bulgaria (BCG-SL 222 Sofia strain, BB-NCIPD Ltd., Bulgaria); and BCG-Danish (BCG-SSI 1331, Statens Seruminstitut, Denmark) [13]. Participants were seen at the clinic for annual visits (at which they gave blood and stool samples and were weighed and measured) and when ill.

Maternal history of TB exposure and disease was ascertained during pregnancy. History of TB exposure and disease in the child was ascertained at annual visits and through illness visits. At one and five years of age, cytokine responses to *Mycobacterium tuberculosis* crude culture filtrate protein (M.tb-cCFP) were assessed. LTBI at age five was assessed using the Interferon gamma release assay (IGRA), T-SPOT.TB^®^ (Oxford Immunotec, Abingdon, UK), for all children in the cohort who turned age five from March 2009 onwards (when the TB sub-study began). Z-scores for weight-for-age, height-for-age and weight-for-height at age five were calculated from World Health Organisation (WHO) growth standards, using WHO Anthro and AnthroPlus macros.

The trial was approved by the Science and Ethics Committee of the Uganda Virus Research Institute, Uganda National Council for Science and Technology, and London School of Hygiene and Tropical Medicine. During pregnancy, women gave written, informed consent for their and their child's participation. The mother, father or guardian gave written informed consent for additional procedures in this study.

### 2.4 Immunological assays

Cytokine responses were assessed among children who had documented BCG immunisation in infancy and who provided a blood sample at five years of age, using a whole blood assay [12,18]. Briefly, unseparated heparinised blood was diluted to a final concentration of one-in-four (RPMI supplemented with penicillin, streptomycin and glutamine), plated in 96-well plates and stimulated with M.tb-cCFP (10 μg/ml; kindly provided by John Belisle, University of Colorado, Fort Collins, USA), tetanus toxoid (TT) (12 Lf/ml; Statens Seruminstitut, Denmark), phytohaemagglutinin (10 μg/ml; Sigma, UK), or left unstimulated. Supernatants were harvested on day six and frozen at −80 °C until analysed. Supernatant cytokine concentrations were measured by Enzyme Linked Immunosorbent Assay (ELISA) (Becton Dickinson, UK). Cytokine production in unstimulated wells was subtracted from concentrations produced in response to stimulation. Cytokine responses were regarded as positive if greater than the higher of the mean plus two standard deviations of the negative control for all assays and the lowest standard in the assay (IFN-γ > 73 pg/ml; IL-5 > 34 pg/ml; IL-13 > 18 pg/ml; IL-10 > 48 pg/ml at one year [18] and IFN-γ >9 pg/ml; IL-5 > 8 pg/ml; IL-13 > 16 pg/ml; IL-10 > 8 pg/ml at five years). Values below the cut-off were set to zero.

Age of infection with cytomegalovirus (CMV) and herpes simplex virus (HSV) were determined by examining for Immunoglobulin G responses by ELISA (DiaSorin, Saluggia, Italy).

To avoid confounding of secular trends with assay performance variability, assays were performed in a randomised sequence after completion of sample collection.

### 2.5 Parasitology and haematology

Stool was examined for helminth ova and Strongyloides larvae using Kato-Katz [28] and charcoal culture [27] methods, respectively. Blood was examined for *Mansonella perstans* using modified Knott's method [29] and for malaria by thick blood film and Leishman's stain. HIV status was determined in mothers and children ≥18 months by rapid antibody test algorithm, and in younger children by polymerase chain reaction [27].

### 2.6 Statistical methods

Data were double-entered into Microsoft Access (Redmond, WA, USA) and analysed using Stata v11 (College Station, TX, USA). The sample size was determined for the trial objectives; from the planned enrolment of 2500 women we expected to retain 1046 children in follow-up at age five [25]. Assuming standard deviation of 0.8log_10_ this sample size would give 80% power to detect a difference in mean cytokine response of 0.14log_10_ for an exposure with prevalence 50%, at 5% significance level.

Outcomes for this analysis were LTBI at age five years determined by T-SPOT.TB^®^ result, and cytokine responses (IFN-γ, IL-5, IL-13 and IL-10) to M.tb-cCFP at age five. Variables considered as exposures were maternal and childhood anthelminthic treatment, maternal socio-demographic characteristics and helminth infections at enrolment; child sex, birth weight, HIV status, illness history, TB exposure/disease, childhood helminth infections, and anthropometry at age five; BCG vaccine strain used for immunisation. In addition, T-SPOT.TB^®^ status was considered as an exposure for age five cytokine responses.

**Fig. 1:**
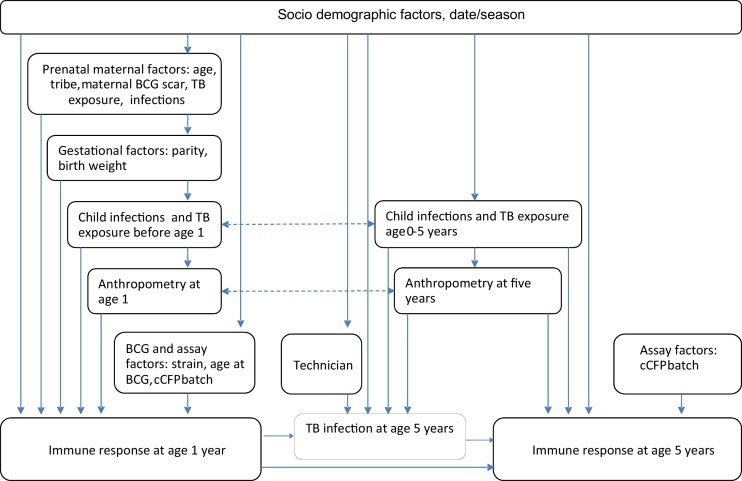
Conceptual framework.

Analyses followed a hierarchical causal diagram approach (Fig. 1) [30]: factors at the same level were considered as potential confounders for each other and for proximal factors. Crude associations were examined and a 15% significance level used to decide which factors to consider in multivariable analyses. Anthropometry variables were not included together in multivariable models to avoid collinearity. Unadjusted effects of trial interventions are reported.

Logistic regression was used to examine associations with LTBI at age five. Cytokine responses were transformed to log_10_ (concentration + 1) and then analysed using linear regression with bootstrapping to estimate bias corrected accelerated confidence intervals [31]; results were back-transformed to give geometric mean ratios.

Spearman's correlation coefficients between cytokines responses at one and five years were calculated. Logistic regression was used to investigate whether cytokine responses at one year were associated with LTBI at five years, restricting to children with history of TB exposure or disease (which implies exposure) between one and five years.

## 3 Results

We enrolled 2507 women resulting in 2345 live births, with 1474 children seen at age five years. Full details on participant enrolment and follow-up are described elsewhere [13,18,26,27]. Of the 1474 children seen at age five, 1191 had documented BCG immunisation in infancy, and cytokine responses to mycobacterial antigen at age five were assessed among this group. For these children, mean maternal age at enrolment was 24 years, 805 (68%) were born to women who were urban residents at enrolment. Ten percent of women reported having been exposed to TB. Hookworm was the most prevalent helminth at 42%; 10% of mothers had asymptomatic malaria. Of the children, 606 (51%) were female; 22 (1.9%) were HIV-infected; 638 (54%) had received BCG-Russia, 445 (37%) BCG-Bulgaria and 107 (9%) BCG-Danish. At age five, mean (SD) weight-for-age, height-for-age and weight-for-height z-scores were −0.9 (0.9), −1.3 (1.2) and −0.1 (1.2), respectively; 63 (5.5%) had asymptomatic malaria, 639 (80.5%) and 691 (98.0%) children had been infected with HSV or CMV, respectively, and 142 (5.9%) had a history of tuberculosis exposure and, or, disease (12 had probable TB disease, as reported by a parent).

At age five, T-SPOT.TB^®^ results were available from 886 children who reached age five years after the TB sub-study began, with 75 infected with tuberculosis (prevalence 8.5%, 95% CI: 6.7–10.5). Of the 1191 children with cytokine results, 1024 (86.0%), 575 (48.3%), 1067 (89.7%) and 849 (71.3%) had detectable IFN-γ, IL-5, IL-10 and IL-13 responses to M.tb-cCFP.

### 3.1 Factors associated with LTBI at age five years

Table 1 shows associations between each exposure and LTBI. Rural residence was associated with reduced odds of LTBI, while history of TB contact or disease was associated with increased odds of LTBI. The odds of LTBI increased with the closeness of the TB contact: compared to contact outside the household, ORs (95% CI) of LTBI for children sharing a living room, a bedroom, and a bed with the contact, were 0.54 (0.13–2.36), 1.79 (0.27–11.86) and 8.33 (1.33–52.03), respectively (trend *p*-value =0.02). There was no evidence for association with LTBI for any other exposure.

### 3.2 Factors associated with cytokine response at age five years

There was moderate positive correlation between cytokine responses at age one and five years for IFN-γ, IL-5 and IL-13 (coefficients 0.20, 0.20 and 0.26, respectively; *p* < 0.001), but no correlation between IL-10 responses (coefficient 0.00, *p* = 0.95).

Crude associations between exposures and cytokine response at age five are shown in Table 2 and exposures remaining associated after multivariable analysis in Table 3. HIV-infected children showed lower IFN-γ and IL-13 responses, compared to HIV-unexposed infants. Asymptomatic malaria at age five was associated with reduced IFN-γ production. There were no consistent associations with maternal helminths or with maternal anthelminthic treatment. In multivariable analyses, childhood helminth infection at any annual visit was not associated with cytokine responses, however, quarterly albendazole treatment during childhood was associated with reduced IFN-γ and IL-13 responses to M.tb-cCFP. Maternal BCG scar was associated with higher IL-10 response to M.tb-cCFP. Greater height-for age was associated with increased responses for all cytokines.

**Fig. 2:**
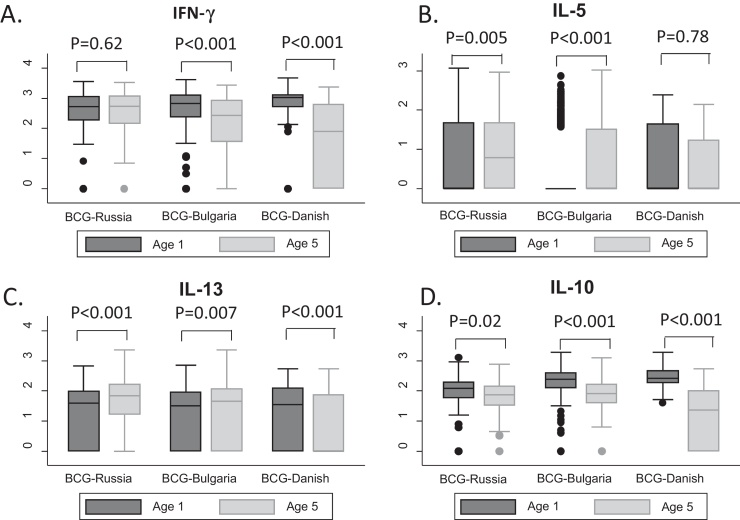
Distribution of cytokine responses to *Mycobacterium tuberculosis* crude culture filtrate protein at age one and five years, by BCG vaccine strain received in infancy. Graphs show distributions of cytokine responses (panel A: IFN-γ, panel B: IL-5, panel C: IL-13, panel D: IL-10) to *Mycobacterium tuberculosis* crude culture filtrate protein at age one year (indicated in blue) and age five years (indicated in pink), separately for children who received BCG-Russia, BCG-Bulgaria and BCG-Danish-values for comparison of responses at age one with responses at age five for each BCG strain and cytokine, were calculated using Wilcoxon signed rank test. (For interpretation of the references to color in this figure legend, the reader is referred to the web version of this article.)

Vaccination with BCG-Danish was associated with lower responses to M.tb-cCFP for all cytokines and BCG-Bulgaria with lower IFN-γ and IL-13 responses, compared to BCG-Russia. IFN-γ responses had waned between age one and five years for children who received BCG-Bulgaria or BCG-Danish, but had remained similar for children who received BCG-Russia (Fig. 2). For the type 2 cytokines, responses among children who received BCG-Russia or BCG-Bulgaria increased between one and five years, while among children who received BCG-Danish they had stayed the same (IL-5) or waned (IL-13).Responses to IL-10 had decreased substantially among children receiving BCG-Bulgaria or BCG-Danish, with a smaller reduction seen among children receiving BCG-Russia (Fig. 2).

Positive T-SPOT.TB^®^ at age five years was associated with higher IFN-γ and IL-13 response to M.tb-cCFP.

Among 58 children with a history of TB contact who had cytokine and T-SPOT.TB^®^ data available, there was no evidence of association between cytokine responses to M.tb-cCFP at one year and odds of LTBI at five years of age (Table 4). We also found no evidence of a relationship between child's BCG scar and T-SPOT.TB^®^ in this subgroup (OR = 1.08, 95% CI: 0.34-3.42, *p* = 0.89).

## 4 Discussion

In this study, using data from a large birth cohort in Entebbe, Uganda, only urban location of residence and history of TB contact or disease during childhood were associated with LTBI at five years. Children testing positive for LTBI at five years had increased type 1 and type 2 cytokine responses to mycobacterial antigens. Other determinants for cytokine response at five years were HIV infection, anthropometry, malaria infection, quarterly albendazole treatment during childhood, maternal BCG scar and the BCG vaccine strain used to immunise the child during infancy.

LTBI prevalence among Ugandan children aged five years was 9% using T-SPOT.TB^®^, comparable with the 12% prevalence among children from Tanzania assessed by QuantiFERON(R)-TB assay [32] but less than the 21% prevalence reported among Ethiopian children [33]. Urban location of residence and history of TB contact/disease were positively associated with LTBI, with odds of LTBI increasing with the closeness of the TB contact. In contrast to findings from Ethiopia, we did not find that helminth exposure, either prenatally, or in childhood, was associated with LTBI [33].

In the same cohort, we have previously investigated factors associated with cytokine responses to M.tb-cCFP at age one [13,18]. Consistent with these findings, HIV infection remained associated with large reductions in IFN-γ and IL-13 responses at age five, and concurrent asymptomatic malaria infection remained associated with reduced IFN-γ response. These results emphasise the importance of HIV infection as a suppressor of immune responses. Since there were no consistent patterns for HIV-exposed-uninfected children, it appears that exposure to HIV *in utero* has no long-term impact on subsequent immune response, as long as the child remains uninfected (despite short-term differences observed in other studies [34,35]).

Anthropometry measures were consistently associated with higher cytokine responses, and these associations were stronger than those observed at one year, indicating that poor growth may have increasing importance among older children.

Consistent with our findings at one year [18], maternal helminth infections were not associated with cytokine responses at age five, suggesting that contrary to some hypotheses [36], maternal helminth infection during pregnancy does not explain poor BCG efficacy in the tropics.

As at one year, maternal BCG scar was associated with cytokine responses at age five, although the association pattern had changed: maternal BCG scar was associated with reduced Th2 cytokine responses at age one, but increased IL-10 responses at age five. These findings support the idea that some aspect of maternal exposure, or of maternal immune response, to mycobacteria may influence the infant immune response to mycobacterial antigen.

Cohort children were randomised to quarterly albendazole or placebo between 15 months and five years. Those receiving albendazole had reduced IFN-γ and IL-13 responses compared to those receiving placebo, as previously reported [26]. This is unlikely to be due to worm removal since childhood helminth infection was not associated with increased cytokine responses (in crude analyses, it was associated with reduced responses). These may be chance findings, since no effects were observed on the response to other antigens tested [26] and given the large number of statistical tests undertaken. Indeed, we did not formally adjust for multiplicity, but instead focus on patterns and consistency of results, when discussing our findings.

At five years, BCG vaccine strain was associated with cytokine production, with children who received BCG-Danish showing consistently reduced responses compared to children who received BCG-Russia, and children who received BCG-Bulgaria showing reduced type 1 and 2 responses, but increased regulatory (IL-10) responses. This implies that the choice of BCG vaccine has a direct effect on the immune response elicited, a hypothesis that is also supported by findings from other studies which have examined the association between BCG strain and immune response during infancy [37,38]. Differences in vaccines are due to evolution from the original BCG vaccine [14,39], for example BCG-Bulgaria is genetically derived from BCG-Russia. “Early” BCG vaccines, such as BCG-Russia, have been hypothesised to have better efficacy than later vaccines [39], although we saw little evidence for this in relation to LTBI, and a recent systematic review found little evidence of a relationship between BCG strain and subsequent efficacy [40].

These findings contrast with those we reported at one year, where infants who received BCG-Danish had markedly higher responses [13]. One possible explanation is that BCG-Danish, initially more immunogenic, could protect against TB infection and possibly against other non-TB mycobacteria, thus children immunised with BCG-Danish are now less likely to have acquired the infections that are the strongest inducers of responses at five years. However, we found no evidence that LTBI status modified the association between BCG vaccine strain and cytokine response to support this hypothesis. Second, studies have shown that the magnitude of cytokine response to mycobacterial antigen gradually reduces over time [3,41]. We found that the decline in cytokine response varied with BCG vaccine strain, with IFN-γ and IL-10 responses among children who received BCG-Danish or BCG-Bulgaria waning faster than among children who received BCG-Russia. Type 2 responses increased among children who received BCG-Russia or BCG-Bulgaria, but stayed the same or decreased among children who received BCG-Danish.

We found no evidence that immune response to mycobacterial antigen at one year was associated with subsequent LTBI, among those with documented exposure to TB disease, although power for this analysis was limited due to the relatively low LTBI prevalence. Further follow-up of the cohort is planned to investigate this. It remains a priority to identify reliable bio-markers of protection following immunisation, if we are to develop a better vaccine against TB.

Our study population exhibited high-prevalence, low-intensity helminth burden in mothers, but low prevalence in children, presumably related to mass drug administration and urbanisation. Findings might differ in populations with continuing high helminth prevalence and in non-TB endemic areas.

In conclusion, prenatal exposure to maternal helminth and malaria infections are unlikely to explain poor BCG vaccine efficacy in the tropics. Concurrent HIV or malaria infection and poor growth are important predictors of immune response. Policies aimed at prevention of mother-to-child HIV transmission and poor growth may contribute to effective immunisation programmes in the tropics. Only urban location of residence and having a history of TB contact/disease were associated with LTBI at five years in this cohort, and it remains to be seen whether the factors we have identified as important for cytokine responses at one and five years will be associated with TB infection at a later age.

## Funding

This work was supported by the Wellcome Trust (063693 and 079110); mycobacterial antigens were provided through the National Institutes of Health contract NOI-AI-25147; albendazole and matching placebo were provided by GlaxoSmithKline. The funders had no further role in the conduct of the research, the preparation of this article, or the decision to submit for publication.

## Contributors

A. Elliott conceived and designed the study. S. Lule conducted the statistical analysis under the supervision of E. Webb. P. Mawa and D. Kizito contributed to sample processing and conducted the cytokine assays. S. Lule, M. Nampijja and F. Akello contributed to recruitment and follow-up of participants and to clinical care. G. Nkurunungi conducted T-SPOT.TB^®^ assays. L. Muhangi was responsible for data management. S. Lule, A. Elliott and E. Webb drafted the report with contributions from G. Nkurunungi, M. Nampijja and L. Muhangi. All authors have read and approved the final article.

## Conflicts of interest statement

The authors have no associations that might pose a conflict of interest.

## Figures and Tables

**Table 1:** Factors associated with T-SPOT. TB among five year old children vaccinated with BCG in infancy.

Factor^a^	TB infection		Crude OR (95% CI)	*P*	Adjusted OR (95% CI)b,c	*P*
	Positive (%)	Negative (%)				
Socio-demographic characteristics						
Maternal age at enrolment			1.02 (0.98–1.06)	0.27		
Parity			1.11 (0.99–1.26)	0.08	1.10 (0.95–1.27)	0.21
Maternal education						
None/primary	34 (7.4)	425 (92.6)	1			
Secondary/tertiary	41 (9.7)	384 (90.4)	1.33 (0.83–2.15)	0.23		
Household SES			0.92 (0.76–1.12)	0.41		
Household crowding			1.14 (0.96–1.36)	0.14	1.03 (0.83–1.27)	0.80
Location of residence						
Urban	59 (10.5)	505 (89.5)	1		**1**	
Rural	16 (5.1)	300 (94.9)	0.46 (0.26–0.81)	0.01	**0.39 (0.19–0.78)**	**0.01**
Maternal characteristics						
*Schistosoma mansoni*						
No	58 (8.3)	645 (91.8)	1			
Yes	17 (9.6)	161 (90.5)	1.17 (0.67–2.07)	0.58		
Hookworm						
No	45 (8.6)	478 (91.4)	1			
Yes	30 (8.4)	328 (91.6)	0.97 (0.60–1.57)	0.91		
*Mansonella perstans*						
No	59 (8.4)	644 (91.6)	1			
Yes	16 (8.7)	167 (91.3)	1.05 (0.59–1.86)	0.88		
Malaria						
No	70 (8.9)	717 (91.1)	1			
Yes	4 (4.8)	79 (95.2)	0.52 (0.18–1.46)	0.21		
Maternal TB exposure						
No	65 (8.2)	723 (91.8)	1			
Yes	9 (9.8)	83 (90.2)	1.21 (0.58–2.51)	0.62		
Maternal BCG scar						
No	27 (7.5)	332 (92.5)	1			
Yes	46 (9.3)	449 (90.7)	1.26 (0.77–2.07)	0.36		
Child characteristics						
Sex						
Male	30 (7.9)	350 (92.1)	1			
Female	36 (9.9)	328 (90.1)	1.28 (0.77–2.13)	0.34		
Birth weight			1.03 (0.59–1.82)	0.91		
HIV status						
Unexposed	64 (8.0)	737 (92.0)	1			
Exposed-uninfected	9 (12.9)	61 (87.1)	1.70 (0.81–3.58)			
Infected	2 (15.4)	11 (84.6)	2.09 (0.45–9.65)	0.30		
BCG scar						
No	29 (8.6)	307 (91.4)	1			
Yes	41 (9.5)	391 (90.5)	1.11 (0.67–1.83)	0.68		
BCG vaccine strain						
Russia	53 (10.1)	471 (89.9)	1		1	
Bulgaria	13 (5.2)	237 (94.8)	0.49 (0.26–0.91)		0.73 (0.35–1.51)	
Danish	6 (6.7)	84 (93.3)	0.63 (0.26–1.52)	0.05	2.25 (0.82–6.22)	0.17
History of TB contact/disease						
No	60 (7.5)	740 (92.5)	1		**1**	
Yes	15 (17.4)	71 (82.6)	2.61 (1.41–4.82)	<0.01	**2.16 (1.02–4.55)**	**0.04**
Any helminth infection ≤5 years						
No	63 (8.8)	652 (91.2)	1			
Yes	12 (7.0)	159 (93.0)	0.78 (0.41–1.48)	0.45		
WHZ score, age 5			0.95 (0.77–1.18)	0.67		
HAZ score, age 5			1.19 (0.96–1.49)	0.11	1.10 (0.86–1.40)	0.44
WAZ score, age 5			1.15 (0.86–1.53)	0.36		
Asymptomatic malaria, age 5						
No	73 (8.9)	752 (91.1)	1			
Yes	1 (2.9)	34 (97.1)	0.30 (0.04–2.25)	0.24		
Number of malaria events			0.96 (0.86–1.07)	0.46		
Number of diarrhoea events			1.03 (0.96–1.12)	0.40		
Number of LRTI events			1.12 (0.92–1.36)	0.26		
Age of infection with CMV			0.73 (0.43–1.23)	0.23		
Age of infection with HSV			1.19 (0.91–1.55)	0.22		
Trial interventions						
Maternal albendazole treatment						
No	39 (8.8)	406 (91.2)	1			
Yes	36 (8.2)	405 (91.8)	0.93 (0.57–1.49)	0.75		
Maternal praziquantel treatment						
No	35 (7.6)	426 (92.4)	1			
Yes	40 (9.4)	385 (90.6)	1.26 (0.79–2.03)	0.33		
Childhood albendazole treatment						
No	37 (8.2)	414 (91.8)	1			
Yes	38 (8.8)	395 (91.2)	1.08 (0.67–1.73)	0.76		

a Missing values: maternal education 2; household socioeconomic status 16; crowding 2; location of residence 6; *Schistosoma mansoni* 5; hookworm 5; maternal malaria 16; maternal TB contact 6; maternal BCG scar 36; birth weight 146; HIV status 2; child's BCG scar 118; BCG strain 2; WHZ score 104; HAZ score 111; WAZ score 101; asymptomatic malaria at five years 6; illness episodes 2.

b Adjusted for each other and for technician.

c Adjusted ORs for which 95% CIs exclude 1 are highlighted in bold.

**Table 2:** Crude associations with cytokine response to crude culture filtrate proteins of *Mycobacterium tuberculosis* in five year olds who received BCG immunisation at birth.

Factor^1^	IFN-γ		IL-5		IL-13		IL-10	
	Geometric mean^2^	Crude GMR (95%CI)^3^	Geometric mean^2^	Crude GMR (95%CI)^3^	Geometric mean^2^	Crude GMR (95%CI)^3^	Geometric mean^2^	Crude GMR (95%CI)^3^
Socio-demographic characteristics								
Maternal age at enrolment		1.00 (0.98–1.03)		**1.02 (1.00–1.04)**		1.01 (0.99–1.04)		1.01 (0.99–1.02)
Parity		0.98 (0.90–1.05)		1.03 (0.97–1.09)		1.00 (0.93–1.07)		0.98 (0.93–1.03)
Maternal education								
None/primary	169.0	1	5.7	1	25.7	1	50.7	1
Secondary/tertiary	163.3	0.97 (0.74–1.29)	5.7	0.99 (0.79–1.24)	25.5	0.99 (0.76–1.28)	50.6	1.00 (0.84–1.21)
Household SES		0.99 (0.90–1.12)		0.94 (0.86–1.03)		0.97 (0.87–1.07)		0.97 (0.90–1.05)
Household crowding		1.05 (0.94–1.16)		1.03 (0.94–1.12)		1.06 (0.95–1.17)		1.03 (0.95–1.10)
Location of residence								
Urban	197.7	**1**	6.3	**1**	29.2	**1**	51.6	1
Rural	114.3	**0.58 (0.42–0.76)**	4.7	**0.75 (0.60–0.95)**	19.4	**0.66 (0.50–0.87)**	48.0	0.93 (0.75–1.11)

Maternal characteristics								
*Schistosoma mansoni*								
No	171.2	1	5.7	1	25.5	1	51.0	1
Yes	146.4	0.86 (0.58–1.20)	5.9	1.03 (0.97–1.09)	26.9	1.06 (0.77–1.44)	48.7	0.95 (0.73–1.18)
Hookworm								
No	159.2	1	5.9	1	25.8	1	48.9	1
Yes	176.7	1.11 (0.84–1.46)	5.6	0.96 (0.77–1.21)	25.6	0.99 (0.78–1.29)	53.1	1.09 (0.89–1.30)
*Mansonella perstans*								
No	174.4	1	6.2	**1**	26.8	1	52.3	1
Yes	143.1	0.82 (0.57–1.12)	4.6	**0.74 (0.58–0.95)**	22.4	0.83 (0.62–1.16)	43.9	0.84 (0.66–1.04)
Malaria								
No	171.5	1	5.8	1	26.1	1	50.7	1
Yes	119.1	0.69 (0.42–1.06)	5.7	0.98 (0.67–1.41)	23.4	0.90 (0.56–1.39)	49.9	0.98 (0.72–1.29)
Maternal TB exposure								
No	162.3	1	5.6	1	25.2	1	51.3	1
Yes	200.2	1.23 (0.77–1.86)	7.3	1.30 (0.89–1.89)	29.7	1.18 (0.77–1.78)	44.7	0.87 (0.64–1.18)
Maternal BCG scar								
No	153.8	1	6.0	1	24.0	1	42.8	**1**
Yes	177.8	1.16 (0.88–1.53)	5.7	0.95 (0.75–1.21)	27.6	1.15 (0.86–1.49)	55.9	**1.31 (1.06–1.61)**

Child characteristics								
Sex								
Male	151.4	1	5.6	1	26.0	1	52.1	1
Female	184.9	1.22 (0.92–1.61)	5.9	1.07 (0.86–1.32)	25.6	0.98 (0.75–1.27)	49.0	0.94 (0.78–1.14)
Birth weight		1.25 (0.92–1.63)		**1.29 (1.00–1.63)**		1.15 (0.85–1.56)		0.95 (0.77–1.18)
HIV status								
Unexposed	170.6	**1**	5.8	**1**	25.8	**1**	50.3	1
Exposed, uninfected	250.9	**1.47 (0.92–2.16)**	6.9	**1.19 (0.84–1.86)**	41.1	**1.59 (1.09–2.27)**	49.5	0.98 (0.67–1.31)
Infected	10.9	**0.06 (0.03–0.22)**	2.0	**0.34 (0.22–0.76)**	2.3	**0.09 (0.05–0.26)**	65.5	1.30 (0.65-2.25)
BCG scar								
No	170.6	1	5.3	1	23.2	**1**	59.3	**1**
Yes	183.3	1.07 (0.79–1.44)	6.5	1.21 (0.96–1.55)	30.0	**1.29 (1.01–1.72)**	46.9	**0.79 (0.65–0.97)**
BCG vaccine strain								
Russia	266.9	**1**	6.6	**1**	34.9	**1**	52.0	**1**
Bulgaria	122.6	**0.46 (0.35–0.60)**	5.5	**0.84 (0.66–1.07)**	22.6	**0.65 (0.51–0.85)**	63.2	**1.22 (1.01–1.46)**
Danish	36.2	**0.14 (0.08–0.23)**	3.3	**0.50 (0.36–0.76)**	7.3	**0.21 (0.13–0.34)**	16.8	**0.32 (0.32–0.48)**
History of TB contact/disease								
No	168.7	1	5.7	1	25.6	1	51.3	1
Yes	147.9	0.88 (0.48–1.43)	6.2	1.08 (0.71–1.60)	28.5	1.12 (0.70–1.76)	43.0	0.84 (0.58–1.17)
Any helminth infection ≤5 years								
No	179.3	**1**	5.7	1	26.0	1	52.3	1
Yes	122.6	**0.68 (0.45**–**0.96)**	6.0	1.06 (0.82–1.43)	25.1	0.96 (0.69–1.36)	43.7	0.84 (0.64–1.09)
WHZ score, age 5		**0.84 (0.75**–**0.95)**		0.97 (0.87–1.07)		**0.88 (0.78–0.98)**		**0.86 (0.80–0.93)**
HAZ score, age 5		**1.37 (1.20–1.54)**		**1.21 (1.10–1.34)**		**1.28 (1.14–1.43)**		**1.24 (1.14–1.34)**
WAZ score, age 5		**1.17 (1.00–1.39)**		**1.20 (1.05–1.36)**		1.14 (0.97–1.33)		1.08 (0.96–1.20)
Asymptomatic malaria, age 5								
No	174.3	**1**	6.0	1	26.1	1	49.3	1
Yes	82.2	**0.47 (0.23–0.82)**	3.8	0.63 (0.41–1.03)	21.1	0.81 (0.45–1.37)	66.9	1.36 (0.84–1.94)
Number of malaria events up to age 5		0.95 (0.89–1.01)		1.01 (0.96–1.06)		1.00 (0.95–1.05)		**1.03 (1.00–1.07)**
Number of diarrhea events up to age 5		1.00 (0.95–1.04)		1.03 (0.99–1.07)		1.02 (0.98–1.07)		1.02 (0.99–1.06)
Number of LRTI events up to age 5		1.02 (0.88–1.15)		1.03 (0.92–1.15)		1.01 (0.88–1.15)		1.02 (0.92–1.11)
Age of infection with CMV		1.17 (0.95–1.37)		**1.17 (1.00–1.38)**		1.17 (0.97–1.37)		1.09 (0.96–1.20)
Age of infection with HSV		0.99 (0.84–1.13)		0.94 (0.84–1.05)		1.00 (0.87–1.14)		1.00 (0.90–1.10)
T-spot TB								
Negative	140.0	**1**	4.8	**1**	18.8	**1**	40.2	1
Positive	380.3	**2.72 (1.51–4.35)**	9.4	**1.97 (1.14–3.41)**	48.7	**2.59 (1.46–4.14)**	43.7	1.09 (0.70–1.62)
Trial interventions								
Maternal albendazole treatment								
No	153.8	1	5.7	1	25.8	1	50.2	1
Yes	180.6	1.17 (0.89–1.54)	5.8	1.03 (0.82–1.28)	25.8	1.00 (0.79–1.32)	50.9	1.01 (0.83–1.19)
Maternal praziquantel treatment								
No	171.3	1	5.7	1	26.2	1	49.3	1
Yes	162.2	0.95 (0.72–1.23)	5.8	1.01 (0.79–1.22)	25.4	0.97 (0.75–1.26)	51.9	1.05 (0.87–1.25)
Childhood albendazole treatment								
No	194.8	**1**	6.2	1	30.5	**1**	50.6	1
Yes	141.3	**0.73 (0.55–0.98)**	5.3	0.86 (0.68–1.05)	21.6	**0.71 (0.56–0.93)**	50.5	1.00 (0.83–1.20)

^1^ Missing values: maternal education 3; household socioeconomic status 22; crowding 4; location of residence 9; *Schistosoma mansoni* 2; hookworm 2; *Mansonella perstans* 2; maternal malaria 19; maternal TB contact 6; maternal BCG scar 10; birth weight 180; HIV status 3; child's BCG scar 157; WHZ score 158; HAZ score 163; WAZ score 155; asymptomatic malaria at five years 36; illness episodes 3. ^2^ Geometric mean of response concentration + 1. ^3^ GMR = geometric mean ratio, confidence intervals not including one are highlighted in bold.

**Table 3:** Factors associated with the cytokine response to crude culture filtrate proteins of *Mycobacterium tuberculosis* in five year olds who received BCG immunisation at birth, after adjustment for potential confounders.

Cytokine/Factor	Adjusted GMR (95% CI)^a^
Interferon-γ	
Child's HIV status	
Unexposed	1
Exposed, uninfected	1.43 (0.88–2.17)
Infected	0.16 (0.05–0.51)
BCG vaccine strain	
Russia	1
Bulgaria	0.46 (0.33–0.63)
Danish	0.19 (0.10–0.36)
Height-for-age *z*-score, age 5 years	1.21 (1.04–1.41)
Asymptomatic malaria, age 5 years	
No	1
Yes	0.50 (0.22–0.96)
T-spot TB	
Negative	1
Positive	2.05 (1.08–3.41)
Childhood albendazole treatment	
No	1
Yes	0.73 (0.55–0.98)
Interleukin-5	
BCG vaccine strain	
Russia	1
Bulgaria	0.89 (0.68–1.15)
Danish	0.59 (0.40–0.91)
Height-for-age z-score, age 5 years	1.15 (1.03–1.29)
Interleukin-13	
Child's HIV status	
Unexposed	1
Exposed, uninfected	1.58 (1.03–2.32)
Infected	0.13 (0.06–0.55)
BCG vaccine strain	
Russia	1
Bulgaria	0.66 (0.50–0.89)
Danish	0.24 (0.15–0.41)
Height-for-age *z*-score, age 5 years	1.14 (1.01–1.27)
T-spot TB	
Negative	1
Positive	2.10 (1.17–3.63)
Childhood albendazole treatment	
No	1
Yes	0.71 (0.56–0.93)

Interleukin-10	
Maternal BCG scar	
No	1
Yes	1.24 (1.01–1.56)
BCG vaccine strain	
Russia	1
Bulgaria	1.27 (1.01–1.58)
Danish	0.45 (0.28–0.73)
Height-for-age *z*-score, age 5 years	1.22 (1.11–1.32)

a Figures are geometric mean ratios of cytokine production, with bootstrap 95% confidence intervals.

**Table 4:** Associations between cytokine responses to mycobacterial antigens at age one and odds of subsequent infection with *M. tuberculosis* infection by five years of age, among 58 children with a history of tuberculosis exposure.

Cytokine response to cCFP at age 1	Crude odds ratio	*P*	Adjusted odds ratio^a^	*P*
IFN-γ	1.10 (0.58–2.09)	0.76	1.14 (0.55–2.37)	0.72
IL-5	1.56 (0.80–3.05)	0.20	1.88 (0.90–3.93)	0.09
IL-13	1.45 (0.72–2.94)	0.28	1.73 (0.76–3.96)	0.19
IL-10	1.23 (0.48–3.16)	0.65	1.21 (0.45–3.26)	0.71

a Adjusted for zone of residence.
